# Probiotic Use and Self‐Reported Psychological, Cognitive, and Gastrointestinal Outcomes in a Community Sample: A Cross‐Sectional Observational Study

**DOI:** 10.1002/brb3.71834

**Published:** 2026-10-08

**Authors:** Kim Marie Gehrke, Eef Hogervorst, Thomas D W Wilcockson

**Affiliations:** ^1^ School of Sport, Exercise and Health Sciences Loughborough University Loughborough UK

**Keywords:** probiotics, gut–brain axis, cognition, gastrointestinal health

## Abstract

**Background:**

Research on probiotics and health outcomes has produced mixed findings. While the gut–brain axis provides a theoretical framework for probiotics to influence psychological and cognitive health, evidence remains inconsistent, particularly in community samples. This study examined associations between probiotic use and psychological, cognitive, and gastrointestinal outcomes, with particular attention to delivery method (supplement vs. fermented food).

**Methods:**

One hundred and fifty adults (*M* = 25.82, *SD* = 10.99) completed validated measures of depressive and anxiety symptoms, stress resilience, gastrointestinal complaints, cognitive lapses, hazardous drinking, and disordered eating. Group differences were examined using Kruskal‐Wallis tests (unadjusted) and two covariate‐adjusted ANCOVA models (Model 1: age, gender, and physical activity; Model 2: additionally adjusting for prebiotic use and dietary adherence).

**Results:**

In unadjusted analyses, probiotic supplement users reported lower depressive and anxiety symptoms, fewer gastrointestinal complaints, and reduced cognitive lapses than non‐probiotic users. After adjustment, gastrointestinal symptoms remained significantly associated with probiotic use across both models (*p* = 0.034, Model 1; *p* = 0.017, Model 2). Anxiety symptoms and cognitive lapses were significantly associated with probiotic use in Model 1 (*p* = 0.026 and *p* = 0.018, respectively) but attenuated to non‐significance in the fully adjusted model. Stress resilience was non‐significant in Model 1 but reached significance in Model 2 (*p* = 0.035). However, none of these associations survived Bonferroni correction for multiple comparisons across the five primary outcomes (α = 0.01). Fermented food users did not differ from other groups on any outcomes.

**Conclusions:**

In this community sample, unadjusted analyses suggested probiotic supplement use was associated with lower depressive symptoms, anxiety symptoms, gastrointestinal symptoms, and cognitive lapses. After adjustment, gastrointestinal symptoms showed the most consistent association, while anxiety symptoms, cognitive lapses, and stress resilience showed inconsistent, model‐dependent associations. No associations were robust to correction, and all findings should be interpreted as preliminary. Longitudinal and experimental research with larger samples is needed to establish causality and clarify the conditions under which probiotic use may confer health benefits.

## Graphical abstract

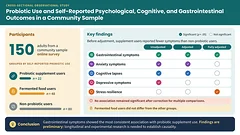

## 1 Introduction

The gut microbiome has long been recognized for its role in brain function, often being referred to as the “second brain” (Stronge and Prudhvi 2024). Meta‐analyses and systematic reviews have demonstrated robust associations between the gut microbiome and multiple domains of health. These include mental health and psychopathology, such as mood‐related outcomes (Shaikh et al. 2025, Makris et al. 2021, Rogers et al. 2016), eating disorders (Lam et al. 2017, Terry et al. 2022), and problematic alcohol use (Bajaj 2019, Leclercq et al. 2014), as well as cognitive functioning (Castells‐Nobau et al. 2024, Tooley 2020), and gastrointestinal health (Trakman et al. 2022, Yadegar et al. 2025). Together, these findings support the gut–brain axis as a multidirectional system through which gastrointestinal functioning may be linked to affective and cognitive processes. Given this close relationship between gut health and broader functioning, there has been increasing interest in interventions that target the gut microbiota.

One of the most accessible approaches for modulating gut microbiota composition is through probiotics. Probiotics are defined as “live microorganisms that, when administered in adequate amounts, confer a health benefit on the host” (Hill et al. 2014). Probiotics have gained traction as potential therapeutic tools across multiple domains due to evidence suggesting benefits for gastrointestinal symptoms (Zeng et al. 2025) and mental health outcomes, including anxiety, depression, stress, disordered eating, and problematic alcohol use (Bahari et al. 2024, Vidya Bernhardt et al. 2024, Dehghani et al. 2023, Merkouris et al. 2024, Yang et al. 2024). A systematic review of recent trials also reported improvements in cognitive function following probiotic supplementation across the lifespan (Fekete et al. 2024), although benefits seem to be more pronounced and consistent among older and cognitively impaired individuals. Collectively, this body of research positions probiotics as a promising strategy for supporting mental health, cognitive functioning, and gastrointestinal health.

Despite these encouraging findings, probiotic research remains marked by substantial heterogeneity, with systematic reviews and meta‐analyses reporting inconsistent or null effects across outcomes (Zeng et al. 2025, McFarland et al. 2023). For example, a recent meta‐analysis found that probiotics generally failed to significantly improve mood, anxiety, depression, or psychiatric distress compared to placebo (Le Morvan de Sequeira et al. 2022), and Bartos et al. reported no benefits for anxiety, depression, or cognitive function in adults (Bartos et al. 2023). While another large population‐based cross‐sectional study did find reduced symptoms of depression following probiotic consumption, these effects were attenuated after covariate adjustment (Cepeda et al. 2017). Such discrepancies are commonly attributed to variation in probiotic strain, dosage, and delivery method, all of which are known to influence efficacy (McFarland et al. 2018, Pot and Vandenplas 2021). However, these factors are often insufficiently detailed or controlled in reviews (McFarland et al. 2023), complicating interpretation of probiotic effectiveness. To this day, the World Gastroenterology Organisation Global Guidelines emphasize that no single generalizable dosage recommendation can be established for probiotics (Guarner et al. 2024). This variability highlights the need for additional research, particularly regarding how probiotics are used in everyday, non‐clinical contexts.

The probiotic delivery method may further contribute to mixed findings. Fermented foods such as yogurt, kefir, kimchi, and kombucha often contain variable and poorly defined microbial strains and, as such, have been argued not to meet the criteria required to be classified as probiotics (Hill et al. 2014). Specifically, to meet the FAO/WHO's definition, probiotics must have defined contents, maintain a viable count through shelf life, and have suitable evidence of health benefits and safety (FAO/WHO 2002). While fermented foods have been associated with improvements in mental health and cognition in some studies (Balasubramanian et al. 2024), they frequently require large and consistent daily consumption to achieve measurable benefits (Ranjha et al. 2021), which may be unrealistic for many individuals. As a result, fermented foods may provide insufficient or inconsistent microbial exposure and would not elicit effects comparable to standardized probiotic supplements. However, direct comparisons of probiotic supplements and fermented foods within naturalistic, community‐based samples remain limited. Moreover, much of the existing research has focused on clinical or diseased populations (Merenstein et al. 2024), with comparatively little attention given to generally healthy individuals seeking preventative or holistic health improvements.

A further limitation of probiotic research is the potential for reverse causality and self‐selection. Individuals with gastrointestinal or psychological symptoms may initiate probiotic use to manage these difficulties, whereas those with better overall health or stronger health‐promoting behaviors may be more likely to adopt probiotics as part of a broader wellness orientation. Evidence from clinical samples suggests probiotic uptake is often symptom‐driven, particularly for gastrointestinal complaints (Lynch et al. 2021), while population‐based associations between probiotic intake and lower depressive symptoms cannot establish directionality (Kim and Shin 2019). Consequently, examining predictors of probiotic use alongside mental health outcomes is essential for clarifying the extent to which observed associations may reflect self‐selection rather than potential benefit.

To further research into probiotics, the present study examines associations between different forms of probiotic use (including probiotic supplements and fermented foods) and a range of self‐reported mental health, cognitive, and gastrointestinal outcomes in a community sample. Potential correlates of any probiotic use are also examined to explore self‐selection. Primary outcomes are mood‐related symptoms (anxiety, depression, and stress resilience), gastrointestinal symptoms, and cognitive lapses, while disordered eating and problematic alcohol use are examined as exploratory mental health outcomes. The study aims to contribute empirically to ongoing debates surrounding probiotic use by evaluating whether the probiotic delivery method is associated with differential outcomes in everyday contexts. Given the observational nature of the study, analyses are intended to be exploratory and hypothesis‐generating.

Given the consistent evidence base linking probiotics to reduced gastrointestinal symptom burden (Zeng et al. 2025), gastrointestinal symptoms were expected to show the most robust association with probiotic use. In contrast, given mixed and frequently null findings for mood‐related outcomes (Le Morvan de Sequeira et al. 2022, Bartos et al. 2023, Cepeda et al. 2017), these associations were expected to be smaller and less consistent. Cognitive lapses were hypothesized to show a modest association, given evidence that cognitive benefits from probiotic supplementation are most pronounced among older and cognitively impaired individuals (Fekete et al. 2024), a population not well represented in this sample. Across all primary outcomes, probiotic supplement users were expected to show lower symptom scores than fermented food users, given the greater variability and lower likely microbial dose associated with fermented food consumption (Hill et al. 2014, Ranjha et al. 2021). Given evidence that gastrointestinal symptoms frequently co‐occur with anxiety and depression (Fond et al. 2014, Söderquist et al. 2020) as well as with poor cognition (Patrick et al. 2023), secondary analyses also examine associations between digestive, psychological, and cognitive outcomes to further contextualize findings within the gut–brain axis framework.

## 2 Materials and Methods

### 2.1 Study Design

This study employed a cross‐sectional observational design to examine associations between probiotic use and psychological, cognitive, and gastrointestinal outcomes in a community sample. Participants were classified into three groups based on self‐reported probiotic use: probiotic supplement users (those reporting current use of a probiotic supplement), fermented food users (those reporting consumption of fermented foods such as yogurt, kefir, kimchi, or kombucha), and non‐probiotic users (those reporting no current use of either probiotic supplements or fermented foods).

### 2.2 Participants and Procedure

A total of 150 participants (58 males, 92 females; *M* = 25.82 years, *SD* = 10.99) were recruited through convenience sampling at Loughborough University and the wider community via online advertisements on social media platforms (Instagram and Snapchat) containing an anonymous survey link. Inclusion criteria required participants to be aged 18 years or older. After providing informed consent electronically, participants completed questions assessing probiotic use and lifestyle factors, followed by a battery of validated self‐report questionnaires. The full survey took approximately 15 min to complete. Ethical approval was obtained from the institutional ethics committee at Loughborough University before study onset (project ID number: 20517).

### 2.3 Measures

#### 2.3.1 Probiotic Use

Probiotic use was assessed using four self‐report items. Participants indicated whether they currently consumed probiotics (yes/no) and the type of probiotic consumed, selecting either “probiotic supplements” or one or more fermented food categories (fermented dairy products, fermented vegetables, fermented soy products, or kombucha). Multiple selections were permitted within the fermented food categories, but participants could not select both the supplement and fermented food options. Participants also reported the frequency of overall use (monthly or less, 2–4 times per month, 2–3 times per week, or daily or almost daily). An open‐ended question asked participants to provide further details regarding probiotic products used, such as probiotic strain, dose, and duration.

Although information on probiotic strain, dosage, and duration of use was requested, responses were highly heterogeneous and frequently incomplete. As a result, these variables were not suitable for reliable quantitative analysis and were not included in statistical models. Participants were therefore categorized based on probiotic delivery method (probiotic supplement users, fermented food users, or non‐probiotic users) for all primary analyses.

#### 2.3.2 Outcome Measures

Participants completed seven validated self‐report questionnaires assessing mental health, cognitive functioning, and gastrointestinal outcomes. Depressive symptoms were assessed using the Patient Health Questionnaire–9 (PHQ‐9) (Kroenke et al. 2001), anxiety symptoms using the Generalized Anxiety Disorder–7 (GAD‐7) (Spitzer et al. 2006), and stress resilience using the Brief Resilience Scale (BRS) (Smith et al. 2008). Gastrointestinal symptoms were measured using the Gastrointestinal Symptom Rating Scale (GSRS) (Svedlund et al. 1988), and cognitive lapses were assessed using the Cognitive Failures Questionnaire–2.0 (CFQ‐2.0; 41), a revised version of the original Cognitive Failures Questionnaire (CFQ) (Broadbent et al. 1982). The CFQ‐2.0 has shown good test‐retest reliability (*r* = 0.88, *p* < 0.001), a factor structure supported by exploratory and confirmatory analyses, and incremental validity over the original CFQ (Goodhew and Edwards 2024). This version was therefore selected for its updated content, demonstrated psychometric improvements, and to reduce participant burden, while acknowledging that its evidence base in community samples remains smaller than that of the original CFQ. Hazardous alcohol use was measured using the Alcohol Use Disorders Identification Test (AUDIT) (Saunders et al. 1993), and disordered eating behaviors and body image concerns were assessed using the Eating Disorder Examination Questionnaire–Short (EDE‐QS) (Gideon et al. 2016).

Depressive symptoms, anxiety symptoms, stress resilience, gastrointestinal symptoms, and cognitive lapses were designated as primary outcomes a priori. Hazardous alcohol use and disordered eating were included as exploratory outcomes.

### 2.4 Covariates and Confounding Variables

Potential covariates were identified a priori based on existing literature (Lee et al. 2018, Dziewiecka et al. 2022, Dai et al. 2023, Tomova et al. 2019, Holscher 2017, Vich Vila et al. 2020, Matenchuk et al. 2020). Participants reported demographic and lifestyle factors, including age, gender (male, female), ethnicity (White, Mixed, Asian, Black, African, or other), physical activity level (sedentary, light, moderate, or vigorous), caffeine intake (none, 1 cup of coffee or matcha, 2–3 cups of coffee or matcha, 4+ cups of coffee or matcha), sleep duration (<6 h, 6–7 h, 8–9 h, or 9+ h), smoking status (no, rarely, sometimes, or yes), prebiotic use (yes/no), and dietary adherence (no, vegetarian, or vegan). Medication use and gastrointestinal conditions were assessed by asking participants whether they had taken any medications within the past three months or had been diagnosed with any gastrointestinal conditions (yes or no). Participants who responded affirmatively were invited to provide additional details via open‐ended responses. Although medication use was analyzed as a binary variable, reported medications included antidepressants, antibiotics, proton pump inhibitors, insulin, beta‐blockers, birth control, ADHD medication, and antihistamines. Visual analysis of these open‐text responses indicated that antibiotics were especially prevalent among fermented food users and antidepressants among non‐probiotic users, while other medication types were approximately equally distributed across groups.

### 2.5 Statistical Analysis

Total scores were calculated for all questionnaires, and BRS scores were averaged in accordance with standard scoring procedures. Data normality was assessed using histograms, Q–Q plots, and Shapiro–Wilk tests, which indicated non‐normal distributions for outcome variables (*p* < 0.05). Accordingly, descriptive statistics for outcome variables were reported as medians and interquartile ranges (IQRs).

Unadjusted group differences across the three probiotic groups were examined using Kruskal–Wallis H tests for each outcome. Where omnibus tests were significant, Bonferroni‐adjusted Mann–Whitney *U* tests (α = 0.0167) were conducted for pairwise comparisons.

To characterize whether candidate covariates differed across probiotic groups, Kruskal–Wallis tests were used for continuous and ordinal variables (age, physical activity level, caffeine intake, and sleep duration). Chi‐square tests were used for categorical variables (prebiotic use, gender, and medication use). Where chi‐square assumptions were violated due to more than 20% of expected cell counts being <5, Fisher's exact tests were conducted (smoking status, ethnicity, gastrointestinal conditions, and dietary adherence). Ethnicity was collapsed into White versus minoritized ethnic background due to sparse cell counts in several categories, to meet assumptions of categorical analyses and improve stability of estimates.

Multicollinearity among predictors included in the primary analyses was initially screened using Spearman's rank correlations and further evaluated using tolerance and variance inflation factor (VIF) diagnostics derived from a linear regression model.

To examine group differences while controlling for covariates, univariate general linear models (GLMs; analysis of covariance [ANCOVA] framework) were conducted for each outcome variable, with the probiotic use group (probiotic supplement users, fermented food users, and non‐probiotic users) entered as the primary independent variable. Two adjusted models were specified: Model 1, which controlled for age, gender, and physical activity level, and Model 2, which additionally controlled for prebiotic use and dietary adherence. Residual normality was assessed for each model using Shapiro‐Wilk and Kolmogorov‐Smirnov (Lilliefors‐corrected) tests, alongside visual inspection of Q‐Q plots and histograms. Where residuals significantly deviated from normality, outcome variables were rank‐transformed and ANCOVA was re‐run on ranked scores, consistent with recommended approaches for non‐normal residuals in general linear models (Conover and Iman 1981). This substantially improved residual distribution across affected outcomes, as reported in the Results. Effect sizes were reported using partial eta squared (ηp^2^) and interpreted according to conventional benchmarks, with values of 0.01, 0.06, and 0.14 reflecting small, medium, and large effects, respectively (Cohen 1988). Covariates were included in adjusted models based on theoretical relevance and parsimony, rather than statistical significance alone. To account for multiple comparisons, a Bonferroni correction was applied across the five primary outcomes (depressive symptoms, anxiety symptoms, stress resilience, gastrointestinal symptoms, and cognitive lapses) tested in the adjusted ANCOVA models, with statistical significance set at α = 0.01.

To evaluate potential confounding and reverse‐causality explanations for probiotic group differences, an exploratory binary logistic regression was conducted to determine whether demographic characteristics, gastrointestinal diagnosis, physical activity, and psychological and gastrointestinal symptom measures predicted probiotic use. Symptom variables were included to assess potential self‐selection into probiotic use rather than to support causal inference. The dependent variable represented any probiotic use (supplement or fermented food) versus no probiotic use. Predictor variables included age, gender, diagnosed gastrointestinal condition, physical activity level, depressive symptoms, anxiety symptoms, gastrointestinal symptom severity, and cognitive lapses. Odds ratios with 95% confidence intervals were estimated.

Spearman's rank‐order correlations were conducted to examine associations among psychological, cognitive, and gastrointestinal outcome variables, due to non‐normal distributions.

All analyses were conducted using SPSS (Version 29.0). Statistical significance was set at *p* < 0.05 unless otherwise adjusted.

## 3 Results

Analyses followed the outcome hierarchy specified in the Introduction. Primary outcomes comprised mood (anxiety, depression, and stress resilience), cognitive, and gastrointestinal domains, while disordered eating and hazardous drinking outcomes were examined as exploratory endpoints.

### 3.1 Descriptive Statistics

Participant characteristics by probiotic group are presented in Table 1. Significant group differences were observed for age, physical activity level, dietary adherence, and prebiotic use (*p* < 0.05), whereas no significant differences were identified for gender, ethnicity, smoking status, sleep duration, caffeine intake, gastrointestinal diagnosis, or medication use (*p* ≥ .05).

**TABLE 1:** Sample characteristics by probiotic use group.

Variable	Probiotic supplement (*n* = 22)	Fermented food (*n* = 48)	Non‐probiotic (*n* = 80)	*p*‐value
Age, years—median (IQR)	25 (9)	23 (3)	22.5 (3)	.005
Gender, n (%)				.307
Male	11 (50)	15 (31.3)	32 (40)	
Female	11 (50)	33 (68.8)	48 (60)	
Ethnicity, n (%)				.895
White	18 (81.8)	37 (77.1)	62 (77.5)	
Minoritized ethnic background	4 (18.2)	11 (22.9)	18 (22.5)	
Smoking status, n (%)				.349
No	11 (50)	33 (68.8)	56 (70)	
Rarely	6 (27.3)	7 (14.6)	7 (8.8)	
Sometimes	2 (9.1)	3 (6.3)	9 (11.3)	
Yes	3 (13.6)	5 (10.4)	8 (10)	
Physical activity level—median (IQR)	2 (1)	3 (2)	3 (1)	.019
Sleep duration—median (IQR)	3 (1)	3 (1)	2 (1)	.454
Caffeine intake—median (IQR)	2 (1)	2 (1)	2 (1)	.178
Dietary adherence, n (%)				.038
No	20 (90.9)	37 (77.1)	74 (92.5)	
Vegetarian	1 (4.5)	10 (20.8)	4 (5)	
Vegan	1 (4.5)	1 (2.1)	2 (2.5)	
Gastrointestinal diagnosis, n (%)	0 (0)	4 (8.3)	3 (3.8)	.356
Prebiotic use, n (%)	13 (59.1)	36 (75)	3 (3.8)	<.001
Medication use, n (%)	3 (13.6)	14 (29.2)	22 (27.5)	.351

*Note*: Values are presented as median (IQR) for continuous or ordinal variables and number (percentage) for categorical variables. *p*‐values represent overall differences across probiotic groups. Kruskal–Wallis tests were used for age, physical activity, sleep duration, and caffeine intake. Fisher's exact tests were used for smoking status, dietary adherence, and gastrointestinal diagnosis due to small expected cell counts. Pearson's χ^2^ tests were used for the remaining categorical variables. Due to small numbers in several ethnic categories, ethnicity was collapsed into White versus Minoritized ethnic background for analysis.

Descriptive statistics for each outcome variable across the three probiotic use groups—probiotic supplement users, fermented food users, and non‐probiotic users—are presented in Table 2. Across primary outcome domains, probiotic supplement users showed descriptively lower symptom scores than fermented food users and non‐probiotic users, with the exception of stress resilience, for which higher scores indicate greater resilience.

**TABLE 2:** Descriptive statistics for outcome measures by probiotic use group.

Outcome	Probiotic supplement (*n* = 22)	Fermented food (*n* = 48)	Non‐probiotic (*n* = 80)
Depressive symptoms	2.50 (4)	4.00 (5)	5.00 (7)
Anxiety symptoms	2.00 (5)	2.50 (7)	5.00 (9)
Stress resilience	3.83 (1.20)	3.59 (1.46)	3.33 (1.25)
Gastrointestinal symptoms	17.00 (9)	21.00 (12)	23.00 (15)
Cognitive lapses	7.50 (15)	11.00 (15)	13.50 (12)
Hazardous drinking	6.50 (6)	5.00 (8)	6.00 (8)
Disordered eating	4.50 (8)	6.00 (11)	6.00 (12)

*Note*: Values are presented as median (IQR).

### 3.2 Group Differences in Primary Outcomes

Kruskal–Wallis tests indicated significant group differences in depressive symptoms, *H*(2) = 7.93, *p* = 0.019, anxiety symptoms, *H*(2) = 8.68, *p* = 0.013, gastrointestinal symptoms, *H*(2) = 7.67, *p* = 0.022, and cognitive lapses, *H*(2) = 9.03, *p* = 0.011. No significant group difference was observed for stress resilience, *H*(2) = 5.15, *p* = 0.076. Bonferroni‐adjusted Mann–Whitney *U* tests (α = 0.0167) indicated that probiotic supplement users reported lower depressive symptoms (*p* = 0.009), lower anxiety symptoms (*p* = 0.011), fewer gastrointestinal symptoms (*p* = 0.008), and fewer cognitive lapses (*p* = 0.002) than non‐probiotic users. No significant differences were observed between probiotic supplement users and fermented food users or between fermented food users and non‐probiotic users for any primary outcomes. Distributions of outcomes showing significant group differences—depressive, anxiety, gastrointestinal symptoms, and cognitive lapses—by probiotic use group are presented in Figure 1. Full Kruskal–Wallis and post‐hoc test results are provided in Supplementary Materials A, Table A1.

**FIGURE 1:**
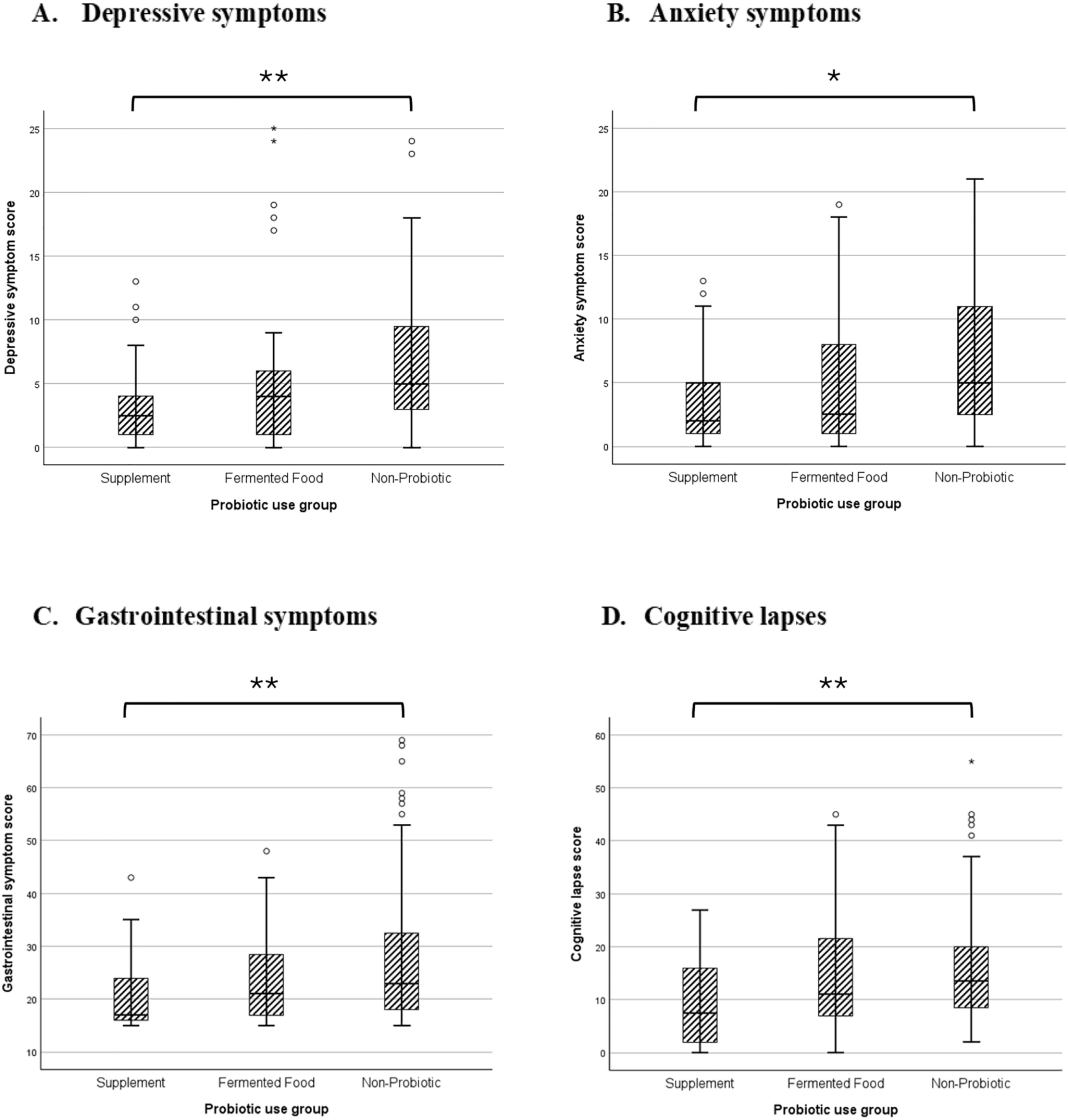
Distributions of depressive symptoms, anxiety symptoms, gastrointestinal symptoms, and cognitive lapses by probiotic use group note. Boxes represent the IQR, horizontal lines indicate medians, whiskers extend to 1.5 × IQR, and points indicate outliers. Probiotic use groups were probiotic supplement users, fermented food users, and non‐probiotic users. Outcomes shown are those with significant unadjusted Kruskal–Wallis tests (*p* < 0.05). Scores represent total questionnaire scale values for each outcome. Brackets indicate significant Bonferroni‐adjusted Mann–Whitney *U* comparisons (α = 0.0167); only supplement vs. non‐probiotic comparisons reached significance. *p < 0.05, **p < 0.01.

### 3.3 Exploratory Analyses

No statistically significant group differences were observed for hazardous drinking, *H*(2) = 1.35, *p* = 0.509, or disordered eating, *H*(2) = 1.23, *p* = 0.540. These mental health outcomes were examined on an exploratory basis and were not designated as primary endpoints.

### 3.4 Covariate‐Adjusted Analyses

Spearman rank correlations among ordinal and continuous covariates indicated no strong associations (all |ρ| < 0.30), suggesting a low risk of multicollinearity. This was confirmed by tolerance and VIF diagnostics from a linear regression model, which showed no evidence of problematic collinearity among predictors, with tolerance values ranging from 0.429 to 0.936 and VIF values ranging from 1.07 to 2.33.

Following rank‐transformation, residual normality improved across all Model 1 outcomes except disordered eating. Depressive and anxiety symptoms no longer significantly deviated from normality (Shapiro‐Wilk *p*s > 0.05). Hazardous drinking was non‐significant under Kolmogorov‐Smirnov despite remaining significant under Shapiro‐Wilk, while gastrointestinal symptoms and cognitive lapses remained significant under both tests. Nonetheless, visual inspection of histograms and Q‐Q plots showed a relatively normal distribution following transformation for these outcomes. Disordered eating showed no improvement under either test and, as a pre‐specified exploratory outcome, results for this outcome should be interpreted with particular caution. A similar pattern was observed for Model 2: Kolmogorov‐Smirnov indicated improved normality for hazardous drinking, depressive symptoms, and gastrointestinal symptoms, while Shapiro‐Wilk remained significant for hazardous drinking, disordered eating, gastrointestinal symptoms, cognitive lapses, and depressive symptoms (borderline, *p* = .045, despite normal Model 1 residuals). Stress resilience residuals were normal in both models and were not rank‐transformed.

In Model 1, significant main effects of the probiotic group were observed for anxiety symptoms, *F*(2, 142) = 3.755, *p* = 0.026, ηp^2^ = 0.050, gastrointestinal symptoms, *F*(2, 142) = 3.472, *p* = 0.034, ηp^2^ = 0.047, and cognitive lapses, *F*(2, 142) = 4.120, *p* = 0.018, ηp^2^ = 0.055, representing small‐to‐medium effect sizes. However, none of these effects remained significant following Bonferroni correction (α = 0.01). No other outcomes reached statistical significance in Model 1 (all *p*s ≥ 0.058).

In the fully adjusted Model 2, a significant group difference remained for gastrointestinal symptoms, *F*(2, 139) = 4.182, *p* = 0.017, ηp^2^ = 0.057, and emerged for stress resilience, *F*(2, 139) = 3.447, *p* = 0.035, ηp^2^ = 0.047, both reflecting small‐to‐medium effects. Neither effect survived Bonferroni correction (α = 0.01). No other outcomes reached statistical significance in Model 2 (all *p*s ≥ 0.091).

To further investigate the pattern observed for stress resilience, a post hoc sensitivity check examined the individual associations between the two covariates added in Model 2 and stress resilience. Prebiotic use was not significantly correlated with stress resilience (*r_s_* = 0.002, *p* = 0.978), whereas dietary adherence showed a weak, significant negative correlation (*r_s_* = −0.205, *p* = 0.012).

Across outcomes demonstrating significant effects prior to correction, adjusted scores were numerically lower among probiotic supplement users relative to non‐probiotic users, with fermented food users typically showing intermediate values. However, as none of these group differences remained statistically significant following correction for multiple comparisons, these patterns should be interpreted as preliminary. Adjusted results for Model 1 and Model 2 are presented in Table 3.

**TABLE 3:** Adjusted group differences in psychological, cognitive, and gastrointestinal outcomes by probiotic use group (Model 1 and Model 2).

	Model 1				Model 2			
Outcome	*F*	*df*	*p*	ηp^2^	*F*	*df*	*p*	ηp^2^
Depressive symptoms	2.909	2, 142	0.058	0.039	1.525	2, 139	0.221	0.021
Anxiety symptoms	3.755	2, 142	0.026	0.050	2.347	2, 139	0.099	0.033
Stress resilience	2.296	2, 142	0.104	0.031	3.447	2, 139	0.035	0.047
Gastrointestinal symptoms	3.472	2, 142	0.034	0.047	4.182	2, 139	0.017	0.057
Cognitive lapses	4.120	2, 142	0.018	0.055	2.443	2, 139	0.091	0.034
Hazardous drinking	0.652	2, 142	0.523	0.009	0.281	2, 139	0.755	0.004
Disordered eating	0.249	2, 142	0.780	0.003	0.466	2, 139	0.628	0.007

*Note*: Model 1 adjusted for age, gender, and physical activity; Model 2 additionally adjusted for prebiotic use and dietary adherence. All outcome variables were rank‐transformed prior to ANCOVA analyses, except stress resilience, which was analyzed on the original scale. No effects met the Bonferroni‐corrected significance threshold (α = 0.01) in either model. *df* = degrees of freedom (effect, error); ηp^2^ = partial eta squared.

### 3.5 Binary Logistic Regression Predicting Probiotic Use

The overall logistic regression model did not significantly predict probiotic use, χ^2^(10) = 12.23, *p* = 0.270. Goodness‐of‐fit indices indicated adequate correspondence between observed and predicted values, χ^2^(8) = 6.77, *p* = 0.562, and the model accounted for a small proportion of variance in probiotic use (Nagelkerke *R* ^2^ = 0.10).

No individual predictor was significantly associated with probiotic use (all *p*s ≥ 0.073). Gastrointestinal symptom severity showed a non‐significant trend toward increased odds of probiotic use, *B* = 0.04, SE = 0.02, *p* = 0.073, OR = 1.04, 95% CI [0.996, 1.09], although the confidence interval included unity. All remaining predictors demonstrated non‐significant associations with probiotic use (all *p*s ≥ 0.270). Full regression coefficients are presented in Supplementary Materials B, Table B1.

### 3.6 Associations between Outcomes

Spearman's rank‐order correlations were conducted to examine associations among psychological, cognitive, and gastrointestinal outcome variables. Depressive symptoms were strongly positively correlated with anxiety symptoms (*r_s_* = 0.81, *p* < 0.01) and moderately positively correlated with gastrointestinal symptoms (*r_s_* = 0.58, *p* < 0.01), cognitive lapses (*r_s_* = 0.53, *p* < 0.01), and disordered eating (*r_s_* = 0.61, *p* < 0.01). Depressive symptoms were moderately negatively correlated with stress resilience (*r_s_* = −0.41, *p* < 0.01).

Anxiety symptoms were moderately positively correlated with cognitive lapses (*r_s_* = 0.58, *p* < 0.01), gastrointestinal symptoms (*r_s_* = 0.57, *p* < 0.01), and disordered eating (*r_s_* = 0.51, *p* < 0.01), and moderately negatively correlated with stress resilience (*r_s_* = −0.52, *p* < 0.01). Gastrointestinal symptoms showed moderate positive associations with five of the six other outcomes, highlighting their centrality within the observed pattern of associations.

Other notable associations included moderate positive correlations between gastrointestinal symptoms and disordered eating (*r_s_* = 0.52, *p* < 0.01) and between cognitive lapses and disordered eating (*r_s_* = 0.43, *p* < 0.01), as well as small positive correlations between hazardous drinking and both cognitive lapses (*r_s_* = 0.18, *p* < 0.05) and disordered eating (*r_s_* = 0.19, *p* < 0.05). Full correlation coefficients are reported in Table 4.

**TABLE 4:** Spearman's rank‐order correlations for study outcome variables.

Variable	Median (IQR)	1	2	3	4	5	6	7
1. Depressive symptoms	4 (6.3)	—						
2. Cognitive lapses	12 (12)	0.53**	—					
3. Gastrointestinal symptoms	21 (12.3)	0.58**	0.51**	—				
4. Stress resilience	3.5 (1.2)	−0.41**	−0.26**	−0.34**	—			
5. Disordered eating	6 (11)	0.61**	0.43**	0.52**	−0.33**	—		
6. Hazardous drinking	6 (6)	0.16*	0.18*	0.13	0.00	0.19*	—	
7. Anxiety symptoms	4 (8.3)	0.81**	0.58**	0.57**	−0.52**	0.51**	0.15	—

*Note. N* = 150. Correlations are Spearman's rho (*r*_s_). * *p* < 0.05, ** p < 0.01.

## 4 Discussion

Previous research on the health impacts of probiotics is characterized by significant heterogeneity and mixed findings. This study examined associations between probiotic use—supplements, fermented foods, or non‐probiotic use—and self‐reported psychological, gastrointestinal, and cognitive outcomes in a community sample. The aim was to determine whether delivery method is linked to differential outcomes in everyday contexts.

### 4.1 Mood‐Related Symptoms

Probiotic supplement users reported lower depressive and anxiety symptoms than non‐probiotic users in unadjusted analyses, consistent with previous research reporting lower anxiety and depression symptom burden following probiotic supplementation (Merkouris et al. 2024). Depressive symptoms did not reach significance in either adjusted model. Anxiety symptoms showed a nominally significant group difference in Model 1, but this effect did not survive Bonferroni correction, and the association attenuated to non‐significance in the fully adjusted Model 2. This pattern of attenuation is broadly consistent with prior work reporting reduced or null probiotic effects on mood‐related outcomes once covariates are accounted for (Cepeda et al. 2017), and with meta‐analytic evidence indicating that probiotic effects on mood‐related outcomes are highly heterogeneous (Le Morvan de Sequeira et al. 2022).

Stress resilience followed a different pattern. Unlike depressive and anxiety symptoms, it did not differ significantly between groups in unadjusted analyses—consistent with evidence that probiotic supplementation tends to show more pronounced effects on depression and anxiety than on stress specifically (Yang et al. 2024)—and remained non‐significant in Model 1. It reached uncorrected significance only in the fully adjusted Model 2, following the addition of prebiotic use and dietary adherence as covariates. This pattern is atypical, as covariate adjustment more commonly attenuates rather than produces significant associations. One possible explanation lies in the distinct roles of the two added covariates. Prebiotic use showed no correlation with stress resilience despite differing significantly by probiotic group (Table 1), consistent with classical suppression, whereby a variable that shares variance with the probiotic group but not the outcome can sharpen the predictor's true association once controlled for. By contrast, dietary adherence was weakly but significantly correlated with stress resilience and also differed by group, suggesting it may instead have masked the group‐resilience association in the unadjusted model, consistent with negative confounding. Together, these patterns offer a plausible statistical account for why the association emerged only after both covariates were added. However, given the small size of the supplement group (*n* = 22) relative to the number of covariates included, this finding may also reflect overfitting rather than a genuine effect and should be interpreted with caution. Consistent with this, the association did not survive Bonferroni correction. Taken together, these mood‐related findings suggest that detectable effects in this sample were inconsistent and may depend on contextual, clinical, or methodological factors rather than reflecting a robust or consistent association.

### 4.2 Gastrointestinal Symptoms

Gastrointestinal symptoms showed the most consistent unadjusted and adjusted association with probiotic supplement use, remaining nominally significant across both adjusted models, in line with prior literature suggesting gastrointestinal benefits are among the more consistently observed outcomes of probiotic use (Zeng et al. 2025). However, as with the mood‐related findings above, this association did not survive Bonferroni correction and should likewise be interpreted as preliminary.

### 4.3 Cognitive Lapses

Cognitive lapses were significantly lower among probiotic supplement users in unadjusted analyses and showed a nominally significant association in Model 1, which was attenuated to non‐significance in the fully adjusted Model 2. Neither adjusted estimate survived Bonferroni correction. This unadjusted pattern is consistent with prior evidence that probiotic supplementation improves cognitive function in patients with mild cognitive impairment and Alzheimer's disease, with more mixed results in younger, healthier individuals (Fekete et al. 2024). Given the present sample's relatively young mean age and correspondingly low median cognitive lapses scores across all groups, symptom variability may have been limited, which could help explain both the modest unadjusted effect and its attenuation after adjustment. The attenuation observed after adjustment may further suggest that the smaller unadjusted difference in cognitive lapses is partly accounted for by demographic and lifestyle covariates. However, the study's limited power to detect small effects, as discussed further in the Limitations section, makes it difficult to distinguish genuine attenuation from insufficient sensitivity to detect true effects.

### 4.4 Exploratory Outcomes

No significant group differences were observed for hazardous drinking or disordered eating in either unadjusted or adjusted analyses. As these outcomes were designated exploratory rather than primary a priori, this null pattern is not unexpected and may reflect genuine absence of association, insufficient statistical power, or the relatively low prevalence of clinically significant symptoms in a general community sample.

### 4.5 Fermented Food Use

No significant differences were observed between fermented food consumers and either probiotic supplement users or non‐probiotic users across any outcome. This lack of effect is consistent with the variability in microbial composition and dosage documented in fermented foods (Hill et al. 2014), which likely explains why fermented food users showed no measurable benefit relative to non‐probiotic users in the present sample. Supporting this, a recent review by Balasubramanian et al. found mixed evidence regarding the relationship between fermented food intake and improvements in mental health and cognitive function (Balasubramanian et al. 2024). One contributing factor may be that fermented foods generally require large and consistent daily consumption to produce measurable benefits (Ranjha et al. 2021), which may not be feasible or typical in community samples. Thus, inconsistent exposure, rather than ineffectiveness, likely explains the null findings among fermented food users.

### 4.6 Healthy‐User Bias and Residual Confounding

The healthy‐user bias explanation, while plausible, requires more specific grounding in the present data. Table 1 shows that probiotic use groups differed significantly at baseline in physical activity level, prebiotic use, and dietary adherence. Notably, fermented food users were more likely to follow a specific diet than probiotic supplement users and non‐probiotic users, and had the highest rate of prebiotic use, while probiotic supplement users did not show a consistent pattern of greater health‐conscious behavior across these variables, and in fact, reported the lowest median physical activity level of the three groups. This suggests that self‐selection into probiotic use is empirically evident in this sample, consistent with evidence linking probiotic and prebiotic use to broader health‐promoting behaviors (Khalesi et al. 2021). However, the present findings suggest this association takes a different form among fermented food users than probiotic supplement users. Groups also differed significantly in age at baseline (Table 1). While age was included as a covariate in all adjusted models, baseline age differences may still reflect broader cohort‐level differences in lifestyle or health awareness not fully captured by statistical adjustment alone. Although models adjusted for key demographic and lifestyle factors and multicollinearity screening confirmed covariate independence, residual confounding from unmeasured factors cannot be ruled out.

### 4.7 Associations Among Outcomes

Correlational analyses showed strong associations between gastrointestinal symptoms and psychological and cognitive outcomes, consistent with gut–brain axis models linking gastrointestinal symptom burden with anxiety, depression, and cognitive functioning (Makris et al. 2021, Rogers et al. 2016, Fond et al. 2014, Söderquist et al. 2020, Patrick et al. 2023). Importantly, these relationships were observed across probiotic use groups, highlighting the central role of gastrointestinal symptoms independent of probiotic use. However, reverse causality remains a central challenge in cross‐sectional probiotic research, as individuals experiencing gastrointestinal symptoms may initiate probiotic use—a pattern supported by clinical data showing that probiotic uptake is often symptom‐driven in gastroenterology settings (Lynch et al. 2021). Interestingly, several lifestyle and dietary variables known to influence gut health did not emerge as significant predictors in adjusted regression models, weakening a strong reverse‐causality explanation for the adjusted gastrointestinal findings. However, this may reflect limited measurement sensitivity, restricted variability, or insufficient statistical power within the present sample.

The exploratory logistic regression model combined supplement and fermented food users into a single ‘any probiotic use’ category, as the analysis was designed to evaluate potential confounding and self‐selection effects, rather than to compare predictors of probiotic use type. As such, this analysis cannot speak to whether different factors predict supplement versus fermented food use specifically, and this remains an open question for future research using larger, purpose‐designed samples.

### 4.8 Limitations

Several limitations should be considered when interpreting these findings. The cross‐sectional design precludes causal inference, leaving open the possibility that individuals with better psychological or gastrointestinal health are more likely to consume probiotics. Indeed, population‐level evidence links probiotic food consumption with lower depression severity and prevalence (Kim and Shin 2019), and, as discussed above, baseline differences in physical activity, prebiotic use, and dietary adherence provide direct evidence of self‐selection in the present sample. Recruitment via social media, particularly Instagram, may have also introduced selection bias, as users of these platforms tend to skew younger, more educated, and more health‐conscious than the general population, potentially limiting the generalizability of these findings to broader community samples. Additionally, probiotic strain, dosage, and duration were not systematically quantified, limiting evaluation of dose–response relationships. This also means the probiotic supplement user group was likely heterogeneous, potentially combining, for example, participants taking a daily, strain‐specific supplement with those using an unspecified product only occasionally. As such, “probiotic supplement use” should be interpreted as a general behavioral category rather than a standardized exposure. This reflects a broader limitation within the literature, where inconsistent reporting complicates comparison across studies (McFarland et al. 2023). Self‐reported prebiotic use also differed substantially across groups, raising the possibility that observed associations may reflect broader microbiome‐targeting health behaviors rather than probiotic use specifically. Furthermore, self‐report measures may introduce potential recall and social desirability biases.

In addition, an a priori power calculation was not conducted, as the anticipated distribution of participants across probiotic groups could not be reliably estimated in advance. In particular, the smaller‐than‐expected probiotic supplement group (*n* = 22) may partly reflect the study's recruitment within a university community with a high proportion of competitive athletes, who are often subject to anti‐doping restrictions that limit supplement use. Consequently, subgroup comparisons—particularly between supplement and fermented food users—were likely underpowered. A post hoc sensitivity analysis based on the fully adjusted ANCOVA model (three groups, five covariates, α = 0.05, 80% power) indicated that the present sample (*n* = 150) was powered to detect effects of approximately *f* = 0.24 (ηp^2^ ≈ 0.055) or larger. Accordingly, smaller effects, particularly for psychological outcomes, may have gone undetected. Additionally, although Bonferroni correction was applied to reduce the risk of Type I error across the five primary outcomes, this is a conservative approach given the moderate correlations observed among outcomes (see Table 4). This may have increased the risk of Type II error, particularly for the underpowered supplement subgroup. Null findings should therefore be interpreted cautiously, highlighting the need for larger‐scale and more controlled investigations, ideally informed by pilot data or stratified recruitment strategies, to clarify the magnitude and specificity of probiotic‐related effects.

### 4.9 Future Directions

Future research should employ longitudinal and experimental designs to establish temporal directionality and causal inference, incorporate microbiome sequencing to verify strain‐specific effects, and collect detailed strain and dosage data to support dose–response modelling. Direct comparisons between probiotic supplements and fermented foods should include microbial verification and quantified intake, given that fermented products do not necessarily meet formal probiotic criteria (Hill et al. 2014). Incorporating objective measures, such as standardized cognitive testing or biological markers of gastrointestinal and psychological functioning, would also help reduce reliance on self‐report and address potential recall and social desirability biases. Larger and clinically diverse samples are also needed to determine whether associations differ among individuals with elevated baseline gastrointestinal or psychological symptoms.

## 5 Conclusions

In conclusion, in this community sample, unadjusted analyses suggested associations between probiotic supplement use and lower depressive symptoms, anxiety symptoms, gastrointestinal symptoms, and cognitive lapses, with gastrointestinal symptoms and, to a lesser extent, anxiety symptoms and cognitive lapses also showing nominally significant associations after covariate adjustment. Stress resilience showed no unadjusted association but emerged as significant only in the fully adjusted model. However, none of these associations survived correction for multiple comparisons and should be interpreted as preliminary. The strong and wide‐ranging correlations observed between gastrointestinal health and other outcomes underscore the relevance of the gut–brain axis and suggest that strategies targeting digestive health may have broader implications for well‐being, though this remains to be tested directly. At the same time, the absence of effects for fermented food users underscores the complexity of real‐world probiotic use, and larger, longitudinal, mechanistic, and strain‐specific research is needed to clarify whether these associations reflect genuine causal pathways.

## Author Contributions

K.M. G. conceived and designed the study, collected the data, performed the statistical analyses, interpreted the results, and drafted and wrote the final version of the manuscript. E. H. reviewed the first draft of the manuscript and provided writing and statistical feedback, and T. W. reviewed and provided feedback on the second draft of the manuscript.

## Funding

The authors have nothing to report.

## Ethics Statement

Ethical approval for this study was granted by the Loughborough University Ethics Committee (Project ID: 20517). All procedures were conducted in accordance with the Declaration of Helsinki.

## Supporting information




**Supplementary Material**: brb371834‐sup‐0001‐SuppMat.docx

## Data Availability

The datasets generated and/or analyzed during the current study are not publicly available due to the inclusion of sensitive participant information, but are available from the corresponding author on reasonable request.
