# Supplementary material for: Probiotic Use and Self‐Reported Psychological, Cognitive, and Gastrointestinal Outcomes in a Community Sample: A Cross‐Sectional Observational Study
Source: Brain Behav. 2026 Oct 8;16(10):e71834. doi: 10.1002/brb3.71834 (PMC13650520; doi:10.1002/brb3.71834)
Supplement: Supplementary file 1 [file BRB3-16-e71834-s001.docx]

**Probiotic Use and Self-Reported Psychological, Cognitive, and Gastrointestinal Outcomes in a Community Sample: A Cross-Sectional Observational Study**

**Supplementary Materials**

**Supplementary Materials A**

**Table A1**

*Kruskal–Wallis H and Bonferroni-Adjusted Mann–Whitney U Test Results Showing Group Differences in Outcomes by Probiotic Use*

| **Outcome** | ***H* (df)** | ***p*** | **Supplement vs Fermented Food (*p*)** | **Supplement vs Non-Probiotic (*p*)** | **Fermented Food vs Non-Probiotic (*p*)** |
| --- | --- | --- | --- | --- | --- |
| Depressive symptoms | 7.93 (2) | **.019** | .268 | **.009** | .077 |
| Anxiety symptoms | 8.68 (2) | **.013** | .478 | **.011** | .030 |
| Stress resilience | 5.15 (2) | .076 | **-** | **-** | **-** |
| Gastrointestinal symptoms | 7.67 (2) | **.022** | .071 | **.008** | .201 |
| Cognitive lapses | 9.03 (2) | **.011** | .068 | **.002** | .230 |
| Hazardous drinking | 1.35 (2) | .509 | **-** | **-** | **-** |
| Disordered eating | 1.23 (2) | .540 | **-** | **-** | **-** |

*Note***.** Bonferroni-adjusted alpha = .0167. Post hoc comparisons were conducted only for outcomes with significant omnibus tests. Significant values are shown in bold.

**Supplementary Materials B**

**Table B1**

*Binary Logistic Regression Predicting Probiotic Use*

| **Predictor** | ***B*** | **SE** | **Wald** | ***p*** | **OR** | **95% CI for OR** |
| --- | --- | --- | --- | --- | --- | --- |
| Age | −0.003 | 0.017 | 0.033 | .856 | 0.997 | [0.964, 1.031] |
| Male | 0.385 | 0.381 | 1.021 | .312 | 1.469 | [0.697, 3.099] |
| GI diagnosis: Yes | −0.953 | 0.896 | 1.131 | .288 | 0.385 | [0.067, 2.233] |
| Physical activity: Light | −0.598 | 0.911 | 0.430 | .512 | 0.550 | [0.092, 3.281] |
| Physical activity: Moderate | −0.956 | 0.939 | 1.036 | .309 | 0.385 | [0.061, 2.421] |
| Physical activity: Vigorous | −0.559 | 0.980 | 0.325 | .569 | 0.572 | [0.084, 3.904] |
| Depressive symptoms | −0.042 | 0.063 | 0.442 | .506 | 0.959 | [0.848, 1.085] |
| Anxiety symptoms | 0.069 | 0.062 | 1.218 | .270 | 1.071 | [0.948, 1.210] |
| Gastrointestinal symptoms | 0.040 | 0.022 | 3.212 | .073 | 1.041 | [0.996, 1.087] |
| Cognitive lapses | −0.002 | 0.021 | 0.007 | .932 | 0.998 | [0.959, 1.039] |

*Note.* OR = odds ratio; CI = confidence interval. Reference categories were female gender, no gastrointestinal diagnosis, and sedentary physical activity. The overall model was not statistically significant, χ²(10) = 12.23, *p* = .270.
